# A Rare Case of Heterozygous C282Y Mutation Causing Hereditary Hemochromatosis With Acute Pancreatitis

**DOI:** 10.7759/cureus.52584

**Published:** 2024-01-19

**Authors:** Roger F Tonna, Rakahn Haddadin, Humzah Iqbal, Hatim Gemil

**Affiliations:** 1 Internal Medicine, MountainView Hospital, Las Vegas, USA; 2 Internal Medicine, University of California San Francisco, Fresno, Fresno, USA

**Keywords:** rare cause of acute abdominal pain, autosomal recessive genetic disorder, primary iron overload, heterozygous hfe gene mutation, c282y and h63d mutations, heterozygous hemochromatosis, hereditary hemochromatosis, acute pancreatitis

## Abstract

Hereditary hemochromatosis (HH) is the most common autosomal recessive genetic disorder globally for Caucasians. HH is known as an iron metabolism disorder where there is an increase in iron absorption in the body. HH is not localized but a systemic disease; the manifestations of HH include cirrhosis, diabetes mellitus, cardiomyopathy, and pancreatitis. This case is about a 53-year-old female with a past medical history of heterozygous hereditary hemochromatosis who presents to the emergency department with abdominal pain, nausea, and vomiting and was found to have acute pancreatitis. This case report helps signify the importance of identifying and treating symptomatic heterozygous carriers of the HH gene mutation.

## Introduction

Hereditary hemochromatosis (HH) is one of the most common autosomal recessive genetic disorders globally for Caucasians [1]. HH is known as an iron metabolism disorder where there is an increase in iron absorption in the body [2]. The mechanism behind the disease is excessive intestinal absorption of dietary iron, resulting in high iron stores in the body's tissues and organs, causing pathological effects [3]. HH is a systemic disease; the manifestations of HH can include cirrhosis, diabetes mellitus, cardiomyopathy, pancreatitis as well as other conditions where iron builds up [3]. HH primarily results from mutations in the HFE gene, with the C282Y mutation being the most prevalent, which accounts for most HH cases [4]. Our case report explores a compelling instance of hereditary hemochromatosis attributed to only one C282Y gene mutation, shedding light on the clinical manifestations, diagnostic challenges, and treatment considerations in managing heterozygous hemochromatosis. Through this case, we aim to emphasize the significance of early detection and intervention in individuals harboring this pathogenic variant, ultimately contributing to improved patient outcomes and preventing severe complications associated with iron overload.

## Case presentation

Our patient is a 53-year-old female with a medical history of heterozygous hereditary hemochromatosis, pancreatitis, Crohn's disease not on any medication for six months, gastroesophageal reflux disease treated with Nissen fundoplication, bleeding peptic ulcer disease, hypertension, asthma, fibromyalgia, anxiety who presented to the emergency department for abdominal pain, nausea, and vomiting. The patient reported left-sided abdominal pain that began a few days before admission. The patient also reported decreased appetite and weight loss for the past two weeks. Further investigation determined that the patient had acute pancreatitis.

While in the emergency department, the patient's vitals were stable. On a physical exam, the patient had immense periumbilical pain and notably worse pain on palpation in the epigastric area. The remainder of the patient's physical exam was negative for acute abnormalities.

Her lab values at admission are shown in Table 1. All other lab values were within the normal range, including the patient's white blood cell count, blood glucose, blood urea nitrogen, creatinine, and calcium levels.

**Table 1:** Lab values

	Results (reference)
Aspartate aminotransferase	42 U/L (15-37)
Alanine transaminase	18 U/L (12-78)
Lipase	1027 U/L (13-75)
Hemoglobin	13.2 g/dL (12.0-16.0)
Hematocrit	38.7% (37.0-47.0)
Sodium	132 mmol/L (135-145)

**Figure 1:**
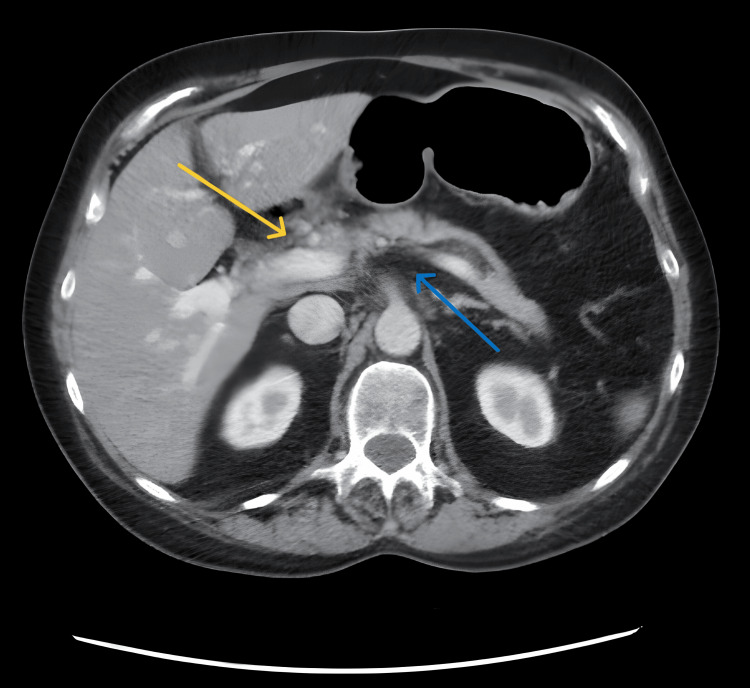
Inflammatory changes seen at the head of the pancreas suggest acute pancreatitis (yellow). Areas of low attenuation with fat stranding are also seen surrounding the pancreas (blue).

A computed tomography (CT) scan of the abdomen and pelvis showed inflammatory changes involving the pancreatic head, likely due to acute pancreatitis. There were some mildly dilated small bowel loops in the upper abdomen and some air-fluid levels, most likely due to a localized ileus related to pancreatitis (Figure 1). An abdominal magnetic resonance imaging (MRI) was performed two days later and showed a re-demonstration of changes in acute interstitial pancreatitis without focal lesions or pancreatic ductal dilatation.

The patient was given ondansetron and metoclopramide as needed for nausea and vomiting and was started on intravenous fluids at presentation. Vitals at the time were temperature 36.8 Celsius, pulse rate 66, respiratory rate 17, blood pressure 123/62, and oxygen saturation 100 on room air. Throughout the patient's time in the hospital, the vital signs stayed within normal limits. After two days of treatment, the patient had no improvement in pain and nausea. Due to the concern of primary sclerosing cholangitis, gastroenterology was consulted, and an esophagogastroduodenoscopy (EGD) was performed. The EGD resulted in a normal esophagus, mild non-erosive antral gastritis, and normal duodenal bulb, but no apparent etiologies were visualized as the cause of her acute pancreatitis.

After six days of being unable to tolerate oral intake, nephrology was consulted to start total parenteral nutrition (TPN). The patient received three days of TPN before tolerating her clear liquid diet.

To look for secondary autoimmune causes, anti-mitochondrial antibodies and immunoglobulins associated with IgG4 were tested, and all came back negative. An erythrocyte sedimentation rate and C-reactive protein were within normal range. Of note, the patient stated that she donates blood every six weeks as part of her treatment for hereditary hemochromatosis.

## Discussion

Primary HH results from two histone family E1 (HFE1) gene mutations, with C282Y and H63D4 most commonly affected [5]. Since HH is an autosomal recessive disease, it is expected that patients affected by the disease should be homozygous for the gene mutation [6]. The most current classification of HH was according to the molecular pattern published in 2022 by BIOIRON, which broke down HH into four subtypes [7]. These subtypes included: 1. HFE-related (C282Y homozygosity, compound heterozygosity, or HFE deletion), in which secondary causes of excess iron should be analyzed and tested for other genetic variants; 2. Non-HFE-related (hemojuvelin HJV-, hepcidin antimicrobial peptide HAMP-, transferrin receptor-2 TFR-2-, and ferroportin 1 SCL40A1-related) in which referral to specialized centers is advised, as is the beginning of phlebotomies; 3. Digenic (homozygosity or heterozygosity in two genes controlling iron homeostasis, either HFE or non-HFE), being challenging to interpret as pathogenic; 4. Molecularly undefined, in which the most common genes are not identified as mutated; thus, a specialized center should take over [8]. According to the above classification, our patient with HH and single allele change in C282Y would fit into the fourth classification. Along with the distinction in classification and manifestations of a homozygous mutation leading to HH, these factors make our presentation rare.

The importance of understanding genetic variants of HH and the allele change is due to the difference in outcome that results. HH with positive HFE predisposes patients, especially males older than 45 years of age, to a more aggressive progression in liver disease that includes fibrosis or cirrhosis, being shown in 25% of them [9]. Homozygous C282Y in males causes a more than ten-fold increase in developing liver carcinoma; guidelines recommend that a patient be monitored every six months via abdominal ultrasound if they are found to have liver cirrhosis [10].

In our patient with heterozygous HH, the patient presented with all of the classic symptoms of acute pancreatitis. Pancreatitis in HH is thought to be caused by iron deposition in the pancreas [11]. HH can also be described as primary (hereditary) or secondary. Primary would be due to mutations in the C282Y, H63D, and S65C loci of the HFE gene, while secondary would be due to multiple blood transfusions or excess dietary iron intake [1]. Two mechanisms can cause HH-induced pancreatitis; in primary hemochromatosis, iron deposition primarily occurs in the liver and pancreas and less often in the reticuloendothelial system (RES), as in the spleen or bone marrow [12]. Effectively imaging iron deposition is done through MRI unless the patient has hepatic steatosis, which can interfere with imaging results, so a unique MRI technique is used [13].

The treatment for hemochromatosis is directed at decreasing the total body iron levels and achieving normal ferritin levels. Therapeutic phlebotomy is usually effective in treating symptomatic HH [14]. Our patient has been receiving constant bloodletting for the past six years. The patient was previously on iron-chelation therapy but did not work nor was she able to tolerate the side effects.

## Conclusions

In conclusion, this case report underscores the significance of heterozygous C282Y mutations in the context of hereditary hemochromatosis (HH). Our findings highlight the critical need for heightened awareness and comprehensive research of carriers of this mutation. While heterozygous carriers typically do not exhibit the severe iron overload observed in homozygous individuals, our case illustrates that they can still experience iron-related complications.

This case serves as a reminder that even heterozygous carriers may require treatment and surveillance to prevent the long-term consequences of iron overload. We strongly advocate for further research into the clinical outcomes and management of heterozygous HH carriers, focusing on refining treatment strategies and surveillance protocols tailored to their needs. Recognizing and addressing the potential risks associated with heterozygous mutations in HH is essential to ensure early diagnosis, appropriate intervention, and improved patient outcomes. This call to action underscores the importance of continued investigation and the development of guidelines for healthcare professionals to better manage and support individuals carrying heterozygous C282Y mutations in hereditary hemochromatosis.
